# Is Alcohol Food?

**Published:** 1862-12

**Authors:** Thomas Inman

**Affiliations:** London, Physician to the London Royal Infirmary


### IS ALCOHOL FOOD?

**By THOMAS INMAN, M. D., London,**

*Physician to the London Royal Infirmary.*

The author first devoted a few words to definition stating that by "alcohol" he intended to comprise those liquors in common use which owed their effects to alcohol; and by "food," anything which supplied food by which the body was nourished. He then adverted to the fact that a saccharine material was found in the blood of all mammals when it entered the lungs, and to the strong probability that a fermentative process took place in those organs, with the extraction of carbonic acid, the actual source of which in the blood had not yet been absolutely ascertained. The close atomic composition of starch and sugar and alcohol *plus* carbonic acid was pointed out; also the fact that the starches, &c., and alcohol were often tolerated by delicate stomachs when other ingredients were not tolerated.

The author then shortly summarized the effects of ordinary food, whether animal or vegetable, when taken with water for a beverage and in proper quantity, and compared these with the results following a temperate draught of ale or porter; showing that there was no real distinction between the one and the other, except that the liquid sooner entered the circulation and sooner left it. It was no argument against the use of beef that a man who had dined on it one day wanted a dinner the day after; nor against beer, that a person who had taken one glass was ready for another in a few hours. The prejudicial effects of excessive eating were adverted to, and after mentioning a few instances where guzzling had proved fatal, others were alluded to in which a prolonged lethargy or an apoplectic condition had been induced. The use of beef-tea sometimes produced convulsions in infants, but this result did not vitiate the dietetic value of meat. The physical condition of excessive eaters was then spoken of, and it was shown that some were thin, others stout; and that as regarded the moral condition of those who, from choice, religious belief, or necessity, abstained from the use of alcoholic beverages, they were to the full as bad as those who indulged in drink. Cannibals were teetotallers, and neither Nana nor Tippo was a drunkard. On inquiring into the habits of total abstainers and those who drank ale, wine, etc., the author had ascertained that the former habitually ate much more than the latter; and one of three deductions was necessary: either the former ate too much, the latter too little, or the drink of the one was equivalent to a portion of the food of the other. To ascertain which of these alternatives was nearest the truth Dr. Inman experimented in his own person, and had made numerous observations through the assistance of friends. The conclusion he came to was that which had been previously insisted on by Mr. Lewes and others,—namely, that alcohol replaced a certain amount of food; and "as things which are equal to the same are equal to one another," he inferred that if a glass of ale was equal to a slice of mutton in its satisfying effect, and that mutton was food, it must follow that ale is food. To say that persons could not live on ale, was of no value as an argument; for no one could live on biscuit alone, though bread was called the staff of life. To ascertain how long it was possible for any one to live on alcohol alone, he had for several years been seeking information respecting drunkards, and he mentioned two—one on the authority of the individual herself (a surgeon's widow), and the other on the authority of the medical attendant, where patients had subsisted for a prolonged period on brandy-and-water alone. He mentioned others on the authority of other medical friends, and two which he had himself been conversant with. He combated the idea of the probability of imposture, inasmuch as in all these cases solid food was loathed excessively, and was generally rejected by the stomach. He then mentioned some cases of children that he had attended, in whom the appetite had failed entirely, where food which was administered by force had been vomited, yet in these alcohol in one form or other gave the support which other food did not, and gradually restored the appetite to its normal state. He noticed, too, that infants at the breast, when ill, would digest brandy-and-water when they would reject all else. The advantageous influence of this fluid was apparent even if it were administered in enemata.

A definite course of induction, irrespective of chemical theory having ended in the conclusion that alcoholic drinks were strictly alimentary, he shortly referred to the statements which were relied upon to demonstrate the contrary. If alcohol, he said, passed out of the system unchanged, so did water; yet water was absolutely necessary to life. But there was no proof that the alcohol imbibed in a long symposium ever left the body. He inferred that if it did pass out of the lungs in vapor as largely as was assumed, a party of spirit drinkers would make the air of a closed room explosive; and he recalled the statement of Percira, that some northern race had found that two or three people in succession might keep up intoxication with "lolium temulentem" by drinking the urine of the first eater; yet none had discovered that the urinal of a drunkard contained anything equal to gin. But certain foods, as oatmeal, bran, potatoes, oats, etc., were not wholly retained in the system, yet they were alimentary.

Dr. Inman then combated the idea that alcohol was a mere stimulant, by contrasting it with turpentine, cantharides, ginger, cayenne, iodide of potassium, and other drugs, which were stimulants to every part of the body to which they were applied. He argued that alcohol could not simply be a conservator of tissue; for a glass of ale after a long walk would induce plentiful perspiration, and a glass of whisky or gin-and-water acted with most people as a powerful diuretic. Nor could we conclude that it assisted in disintegrating the tissues; for if it did, the use of ale, wine, or spirit must then be antagonistic or antidotal to food, and the winebibber must necessarily require more food than the teetotaller, whose tissues were not disintegrated by artificial means.

He then summed up his conclusions thus:

1. Nature has provided in the salivary glands, the liver, and the lungs of every mammal an apparatus for converting all food, especially farinaceous, into alcohol; and we have no evidence that such conversion does not take place.

2. One form of alcohol or another is available for the support of life, and for restoration to health when no ordinary food can be or is digested.

3. Alcohol, after being taken, is incorporated with the blood, passes into the various tissues, and ultimately disappears, a small portion only passing away in the breath. We can say no more of bread, potatoes, or oatmeal porridge, a small portion of each of which passes out of the body with the fæces.

4. Alcohol in the form of ale, porter, wine, etc., relieves hunger and quenches thirst simultaneously, and with a completeness that is not equalled by water, infusion of gentian, cayenne pepper, or by turpentine—i. e., it does not act as water simply, or as a stimulant alone.

5. Wine, beer, etc., satisfy the appetite when taken alone, and act for the time like any solid food would do.

6. When alcohol is mingled with other food, a less amount of the latter suffices for the wants of the system than if water had been used as the drink.

7. The various forms in which alcohol is taken have as marked and specific effects as have animal and vegetable articles of diet.

8. Individuals have subsisted wholly upon one or other of the various forms of alcohol in common use for periods of great length; and as it is illogical to conclude that they lived on air, without food, or on flies like chameleons, the conclusion is irresistible.

What that conclusion is, it might be left for every thinking man to decide.—*London Lancet.*

